# Targeting cell death signalling in cancer: minimising ‘Collateral damage'

**DOI:** 10.1038/bjc.2016.111

**Published:** 2016-05-03

**Authors:** Joanna L Fox, Marion MacFarlane

**Affiliations:** 1 MRC Toxicology Unit, Hodgkin Building, PO Box 138, Lancaster Road, Leicester LE1 9HN, UK

**Keywords:** cell death, cancer, TRAIL, BCL-2 family, IAPs, SMAC mimetics, targeted therapies, adverse effects

## Abstract

Targeting apoptosis for the treatment of cancer has become an increasingly attractive strategy, with agents in development to trigger extrinsic apoptosis via TRAIL signalling, or to prevent the anti-apoptotic activity of BCL-2 proteins or inhibitor of apoptosis (IAP) proteins. Although the evasion of apoptosis is one of the hallmarks of cancer, many cancers have intact apoptotic signalling pathways, which if unblocked could efficiently kill cancerous cells. However, it is becoming increasing clear that without a detailed understanding of both apoptotic and non-apoptotic signalling, and the key proteins that regulate these pathways, there can be dose-limiting toxicity and adverse effects associated with their modulation. Here we review the main apoptotic pathways directly targeted for anti-cancer therapy and the unforeseen consequences of their modulation. Furthermore, we highlight the importance of an in-depth mechanistic understanding of both the apoptotic and non-apoptotic functions of those proteins under investigation as anti-cancer drug targets and outline some novel approaches to sensitise cancer cells to apoptosis, thereby improving the efficacy of existing therapies when used in combination with novel targeted agents.

Clinicians and scientists are continually striving to improve survival rates for cancer patients by searching for novel targets as well as trying to improve existing therapies. Despite the huge array of proteins and biological processes that both targeted and ‘cytotoxic' chemotherapeutic agents perturb, for efficacy, the majority of these agents depend upon the induction of a form of cell death known as apoptosis. Evasion of apoptosis is, however, one of the hallmark traits of cancer (Hanahan and Weinberg, 2011), resulting in high levels of inherent as well as acquired resistance to many chemotherapeutic agents. Targeting apoptotic proteins has therefore become an attractive anti-cancer strategy with numerous points within apoptotic signalling pathways currently under investigation for the treatment of cancer. However, due to the inherent resistance to apoptosis associated with many cancers, much higher doses of drugs are required to achieve efficacy thus increasing the risk of off-target adverse effects.

Apoptosis predominantly proceeds via two distinct routes; the extrinsic and intrinsic apoptosis pathways. The extrinsic pathway is initiated by extracellular pro-apoptotic stimuli including cell death ligands of the TNF cytokine family that act via death receptors located on the cell surface (reviewed in Dickens *et al*, 2012b). Binding of ligands to their cognate receptors results in the formation of the death-inducing signalling complex (DISC), which activates the initiator caspases 8 and 10. Activated caspase 8 can then cleave the effector caspases 3 and 7 to amplify the death signal. The intrinsic pathway, however, is initiated by intracellular pro-apoptotic signals such as DNA damage or exposure to cytotoxic agents. The BCL-2 family of proteins that regulate intrinsic apoptosis senses these death signals resulting in the initiation of mitochondrial (intrinsic) apoptosis. The BCL-2 family consists of anti-apoptotic (including BCL-2, Mantle cell lymphoma-1 (MCL-1) and BCL-xL) and pro-apoptotic (including BIM, BID, PUMA, NOXA and BAD) proteins as well as effector proteins (BAK and BAX). The balance of these different family members determines whether BAK and/or BAX are activated causing mitochondrial outer membrane permeabilisation (MOMP) and release of pro-apoptotic proteins such as cytochrome *c*, facilitating apoptosome formation and activation of caspases 9 and 3. Importantly, there is also crosstalk between the two apoptotic pathways, via caspase 8 cleavage of BID resulting in amplification of the death signal. Downstream of caspase activation there is a further level of regulation by inhibitor of apoptosis (IAP) proteins. X-linked inhibitor of apoptisis (XIAP) is able to inhibit apoptosis by directly binding to and inhibiting caspases 9 and 3, whereas the cellular IAP (cIAP) proteins inhibit caspase activity indirectly via their ubiquitin ligase activity promoting pro-survival signalling.

## Targeting apoptotic pathways

**Figure 1:**
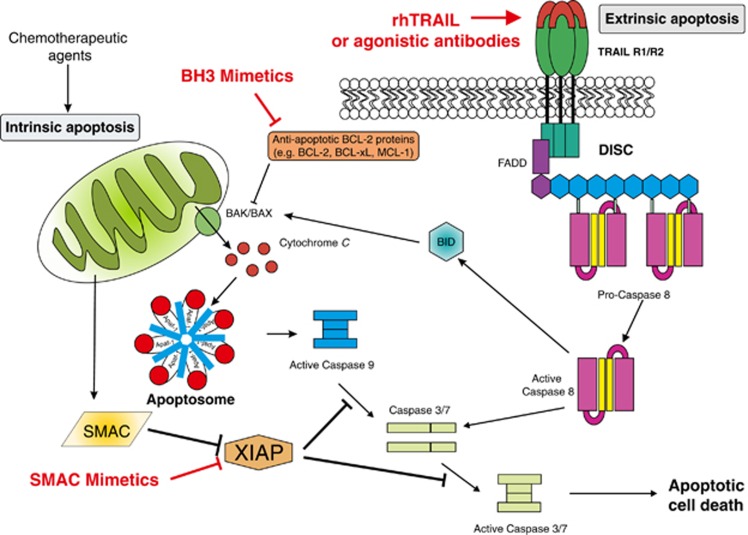
**Intrinsic and extrinsic apoptotic signalling pathways and points of therapeutic intervention.** Apoptosis can be initiated by signals originating from either the plasma membrane via death receptor ligation (extrinsic pathway) or at the mitochondria (intrinsic pathway). Stimulation of the extrinsic pathway by TRAIL results in TRAIL receptor (TRAIL-R) aggregation and formation of the DISC, in which pro-caspase 8 becomes activated and initiates apoptosis by direct cleavage of downstream effector caspases. The addition of either agonistic TRAIL-R1/R2 antibodies or recombinant human TRAIL (rhTRAIL) has been used to trigger the extrinsic pathway for therapy. The intrinsic pathway is regulated by the BCL-2 family of proteins, which regulate pore formation in the outer mitochondrial membrane and release of apoptogenic factors such as cytochrome *c* or SMAC from the mitochondria. The release of cytochrome *c* into the cytosol triggers caspase 9 activation through the formation of the cytochrome *c*/Apaf-1/caspase 9-containing apoptosome complex. SMAC promotes caspase activation through neutralising the inhibitory effect of IAPs. The intrinsic pathway has been targeted for therapy either by blocking the inhibitory action of the pro-survival BCL-2 family proteins with BH3 mimetics or by inhibiting the anti-apoptotic action of IAPs with SMAC mimetics. The extrinsic and intrinsic pathways are interconnected, for example, by BID, a BH3 domain-containing protein of the BCL-2 family, which upon cleavage by caspase 8 triggers intrinsic apoptosis, thereby further amplifying the signal from the extrinsic pathway.

Apoptosis is a complex process with numerous points of regulation, manipulation of which could provide therapeutic benefit in cancer treatment. A greater understanding of these regulatory pathways and how they are altered in cancer, leading to apoptosis resistance, has been key in the development of pro-apoptotic agents. Interestingly, mutation rates in apoptotic proteins such as death receptors (Lee *et al*, 2001) or BCL-2 proteins (Kim *et al*, 2012) are low indicating that many cancer cells have intact apoptotic machinery which if unblocked could efficiently kill tumour cells. Figure 1 illustrates some of the points in apoptotic signalling pathways that have been targeted to overcome the block in apoptosis. These include; triggering extrinsic apoptosis via death receptors by the addition of exogenous ligands such as recombinant human TRAIL (rhTRAIL) or agonistic TRAIL receptor (TRAIL-R1/R2) antibodies. Recombinant human TRAIL binds to both TRAIL-R1 and TRAIL-R2 causing cancer-specific cell killing by initiating DISC formation and apoptosis. Agonistic antibodies, which have a much longer bioavailability than rhTRAIL, specifically target the individual receptors and were found to be more effective at increasing caspase activity and apoptosis (reviewed in Lim *et al*, 2015).

Efforts to initiate intrinsic apoptosis have focused on targeting anti-apoptotic BCL-2 family members. BCL-2 has four BCL-2 homology or BH domains; BH1, 2 and 3 form binding interfaces for protein–protein interactions with other BCL-2 family members required for the initiation of apoptosis. BH4 is the pro-survival domain of the protein as removal of this domain converts BCL-2 from anti- to pro-apoptotic (Han *et al*, 2015). Modulation of the activity of these proteins has been achieved using pro-apoptotic BH3-only protein mimetics, which upon binding to the anti-apoptotic proteins neutralises their activity by sequestering them, enabling apoptosis to proceed. Numerous inhibitors are being developed; however, they have been found to have limited efficacy as single agents in clinical trials (reviewed in Vela and Marzo, 2015); one notable exception is in chronic lymphocytic leukaemia (CLL), where promising results have been reported for ABT-199 as a single agent (Roberts *et al*, 2016). In addition, agonistic mimetic compounds targeting the ‘pro-survival' BH4 domain of BCL-2 have been developed, which are showing promising pre-clinical results (Han *et al*, 2015), but to date no clinical data are available on these compounds.

A third approach has been to focus on regulatory pathways such as increasing caspase activity by inhibiting IAPs using compounds that mimic the endogenous IAP antagonist SMAC. These agents release the inhibitory action of XIAP on caspases, as well as causing ubiquitin-dependent degradation of the cIAP proteins and thus altering NFkB signalling from pro-survival to pro-death. As SMAC is known to form homodimers when binding to IAPs, both monovalent (e.g., LCL161) and bivalent (e.g., BV6 and Birinapant) SMAC mimetics have been developed, with the bivalent compounds showing higher binding affinity and correspondingly higher potency and anti-cancer activity. As single agents these compounds were found to be dependent on the presence of a functional autocrine/paracrine signalling loop involving death receptor ligands and their corresponding receptors for induction of cell death (Petersen *et al*, 2007; Varfolomeev *et al*, 2007; Vince *et al*, 2007). This therefore limits the range of malignancies where these agents would be effective as single agents.

## Double-edged sword: limitations of targeting cell death proteins

Although many of these approaches showed promise in the pre-clinical setting, this has not always been translated to the clinic. Understanding the mechanisms of action of these proteins both in apoptotic and non-apoptotic signalling pathways is therefore essential in order to maximise their clinical usefulness. In this respect, it is often disruption of the non-apoptotic functions of these proteins that result in either on-target adverse effects and/or off-target toxicity.

Efforts to induce extrinsic apoptosis have focused on TRAIL signalling as targeting the death receptors, TRAIL-R1 and TRAIL-R2, resulted in much lower toxicity compared with that observed when either TNF or FAS signalling were targeted. This was in part due to the observation that there were lower expression levels of TRAIL receptors on the surface on normal cells compared with cancer cells, giving rhTRAIL and TRAIL-R1/R2 agonistic antibodies a therapeutic window. Cancers signal predominantly via either TRAIL-R1 or TRAIL-R2, interestingly though the extent of relative surface expression of the two receptors does not always act as an indicator of the preferred receptor for signalling (MacFarlane *et al*, 2005; reviewed in Dyer *et al*, 2007). Although there are generally higher levels of expression of these receptors on cancer cells, there are reports of high levels of expression of TRAIL receptors on hepatocytes, brain tissue and keratinocytes (Jo *et al*, 2000; Leverkus *et al*, 2000). This raised concerns about possible hepatotoxicity when TRAIL was used in the clinical setting, especially as one study reported significant apoptotic cell death in fresh isolated primary hepatocytes (Jo *et al*, 2000), though significantly this effect was not observed under conditions of optimal hepatocyte *in vitro* function (Ganten *et al*, 2006). Moreover, in organotypic culture of fresh healthy liver explants, which more closely mimic the liver in the clinical setting, no toxicity was observed with TRAIL treatment alone (Volkmann *et al*, 2007). Importantly, treatment of diseased liver tissue, however, was much more sensitive to TRAIL treatment resulting in significant cell death upon exposure to TRAIL (Volkmann *et al*, 2007). Conversely, when HDAC inhibitors were combined with TRAIL (a combination that had proved very effective in sensitising CLL cells isolated from patient samples resistant to rhTRAIL (MacFarlane *et al*, 2005)), in the same organotypic culture conditions used for TRAIL treatment alone, significant hepatotoxicity was observed for the combination (Volkmann *et al*, 2007). Notably, a recent clinical trial of a second-generation TRAIL-R2 agonist, TAS266, which enhances receptor clustering, had to be halted due to dose-limiting hepatotoxicity (Papadopoulos *et al*, 2015). Clearly, these findings highlight that care must be taken when selecting appropriate models both for evaluating drug combinations and for identifying potential on-target adverse effects and/or off-target toxicities.

In addition to potential hepatotoxicity, recent reports have further highlighted other non-apoptotic roles for TRAIL signalling, in particular a role in cell proliferation and migration. A study in KRAS mutant cancers, which have high TRAIL-R2 expression, found that stimulation of TRAIL signalling in fact promoted cancer progression, invasion and metastasis. There was a direct correlation between the levels of receptor expression and the extent of metastasis observed in patients (von Karstedt *et al*, 2015). Studies in pancreatic cancer looking at the nuclear role of TRAIL-R2 signalling also revealed that nuclear TRAIL-R2 interacts with the core miRNA processing complex causing an inhibition of maturation of the miRNA, let-7 (Haselmann *et al*, 2014). The consequence of this interaction is an increase in the levels of let-7 target genes that result in tumour progression. Moreover, continuous exposure to sublethal concentrations of TRAIL induces an NFkB-dependent increase in miR-21, miR-30c and miR-100, which downregulates expression of caspase 8, caspase 3, TRAF7 and Foxo3a, resulting in acquired resistance to TRAIL and the development of more aggressive tumours (Jeon *et al*, 2015). Subtoxic doses of TRAIL also induce caspase-8-dependent activation of apoptotic nucleases giving rise to increased mutations caused by misrepair of DNA damage in surviving cells (Lovric and Hawkins, 2010). These studies highlight the unintended consequences that could occur when patients are treated with agents that activate TRAIL signalling.

BH3 mimetics have been the main class of agents targeting the intrinsic pathway. However, once in clinical trials, the orally bioavailable BCL-2/BCL-xL antagonist ABT-263 (Navitoclax) was found to have dose-limiting toxicity of thrombocytopenia, as a result of the induction of platelet death. Further investigation of the mechanism of cell death in platelets treated with pan-BCL-2 inhibitors revealed that in these cells BCL-xL is the critical anti-apoptotic protein required to prevent activation of the effector proteins BAK and BAX. This effect was also demonstrated in numerous mouse knockout models (reviewed in Sochalska *et al*, 2015), in which BCL-xL knockout mice were found to have decreased numbers of circulating platelets, confirming that BCL-xL has a critical role in the development and survival of platelets; the consequence of which, is that ABT-263 can only be used at low doses. Interestingly, Tait *et al* have since reported that the use of these BH3 mimetics at doses used in the clinic may in fact be oncogenic. They demonstrated that incomplete initiation of MOMP by these agents, known as minority MOMP, can result in caspase-dependent transformation and tumourigenesis, which in turn can promote more aggressive, invasive tumours (Ichim *et al*, 2015). In addition, *in vitro* studies to investigate the mechanisms of acquired resistance to BH3 mimetics observed mutations in BCL-2 family proteins following extended exposure to these agents (Fresquet *et al*, 2014). BCL-2 family members are also reported to negatively regulate autophagy; thus, BH3 mimetics have been found to modulate both apoptosis and autophagy induction. Due to the dual role of autophagy in regulating cell death and survival, the induction of autophagy by these agents has been found to be both cytotoxic and protective depending on the model investigated (reviewed in Yu and Liu, 2013).

Due to structural differences in the BCL-2 family members the first BH3 mimetics developed did not target MCL-1, and resistance to these early agents was initially attributed to the fact that MCL-1 may be compensating for the inhibited BCL-2 family members. There are now however the first reports of bona fide MCL-1-specific inhibitors, shown to trigger BAX/BAK-dependent apoptosis (Belmar and Fesik, 2015; Leverson *et al*, 2015). Nevertheless, one question remains as these compounds are investigated, what will be the response of normal tissues to MCL-1 inhibition by these inhibitors? Mouse knockout studies to date report numerous toxic side effects resulting from MCL-1 ablation (Sochalska *et al*, 2015). For example, MCL-1 has been shown to have an essential role in basal myocyte homeostasis and ablation of MCL-1 in adult myocytes results in mitochondrial dysfunction, impaired autophagy and consequently rapid development of heart failure (Thomas *et al*, 2013). However, it will be important to evaluate the differences in the phenotypes observed between complete gene ablation in mouse models compared with pharmacological inhibition with small molecules. MCL-1 is also reported to have roles, depending on its mitochondrial localisation, in control of mitochondrial dynamics and homeostasis (Thomas *et al*, 2013; Varadarajan *et al*, 2013), normal ATP production and the maintenance of mitochondrial membrane potential, cristae ultrastructure, as well as the oligomeric structure of ATP synthase (Perciavalle *et al*, 2012). Inhibition of this key protein therefore may have unforeseen and potentially deleterious consequences.

Downstream of the death receptors and BCL-2 family are the IAP proteins, which are highly expressed in cancers and have been linked to tumour progression as well as poor prognosis and the development of resistance to therapy. However, cIAPs have a second role in the cell regulating the noncanonical NFkB pathway via the NFkB-inducing kinase (NIK). In the absence of cIAPs, there is an upregulation of inflammatory cytokines, such as TNF which is pro-death and so would amplify anti-IAP therapy. However, other cytokines are also increased such as IL-8, IL-6, IL-10 and MCP-1, all of which contribute to a systemic increase in inflammatory cytokines and have also been reported to be involved in tumour progression via cancer-associated inflammation causing the recruitment of immune cells to the cancer site where they support tumour growth. In fact, cytokine release syndrome was determined to be the dose-limiting toxicity in clinical trials of the SMAC mimetic LCL161 (Infante *et al*, 2014) and was also observed, but was not determined to be the rate-limiting toxicity, in the phase 1 dose-escalation study for Birinapant (Amaravadi *et al*, 2015). Interestingly, in the later trial, the pharamacodynamic analysis of patient samples revealed that modulation of the target was achieved and maintained well below the determined maximum tolerated dose. Therefore, one needs to ask whether it is possible to find a balance between effectively modulating the target protein without causing a detrimental inflammatory phenotype. One further caveat to this however is that some studies with yet another SMAC mimetic BV6 observed that, at lower doses, treatment of cells with the compound actually increased migration and invasion of the cancer cells being studied (Tchoghandjian *et al*, 2013). Furthermore, at the same low doses, there was also an increase in the differentiation of cancer stem-like cells (Tchoghandjian *et al*, 2014).

**Figure 2:**
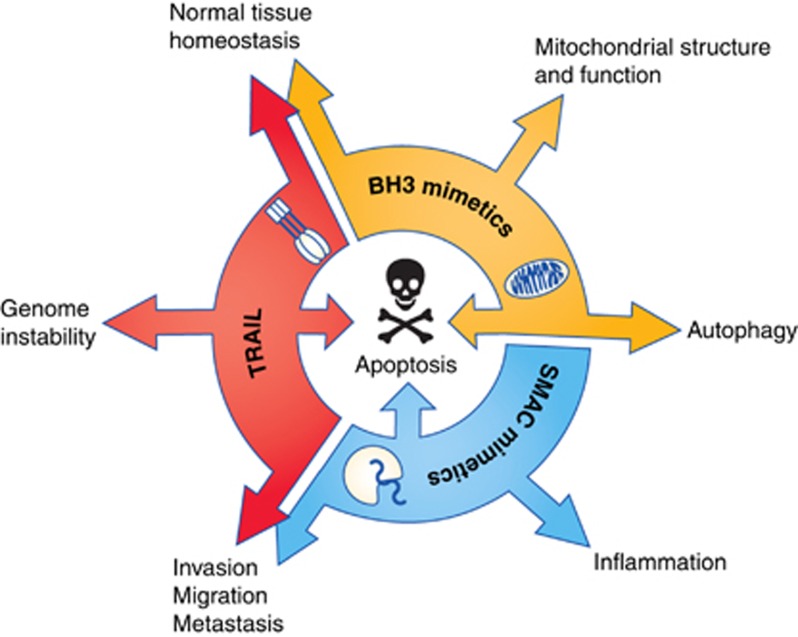
**Limitations of targeting cell death proteins for cancer therapy.** Schematic representation of the additional processes targeted when cell death proteins and pathways are modulated. For example, modulation of TRAIL signalling can cause genome instability and alterations to normal tissue homeostasis. Inhibition of BCL-2 proteins can alter the mitochondrial structure and function, and inhibition of IAPs can result in increased inflammation.

All these examples, summarised in Figure 2, highlight the fact that to target apoptotic pathways a delicate balance must be achieved, to sensitise or lower the apoptotic threshold of tumour cells, without modulating the other pathways where these proteins contribute to making some cancers more resistant to treatment or more aggressively invasive.

## Sensitising cancer cells to die: improving therapeutic outcome

More detailed understanding of the regulation and activation processes of apoptotic pathways is proving to be key in determining new strategies to make existing therapies more effective. By targeting specific blockades in apoptosis signalling, it may be possible to make chemoresistant tumours more sensitive to treatment with existing therapies. Dynamic BH3 profiling to assess how close a cell is to the apoptotic threshold represents one method that enables chemosensitive *vs* chemoresistant tumours within the same histology to be identified (Montero *et al*, 2015). The level of priming of a cell before therapy has been found to be an excellent predictor of chemotherapeutic response *in vivo* (Ni Chonghaile *et al*, 2011; Vo *et al*, 2012). However, for those ‘unprimed' tumours that are resistant to conventional chemotherapeutic agents, alternative approaches are required to increase their level of ‘priming' and make them more sensitive to treatment.

Extensive mechanistic studies have been undertaken to understand the precise mechanism of the conversion of pro-caspase 8 to the mature molecule, which can be activated and initiate apoptosis. In particular, it has been shown that stable pro-caspase 8 DED chains must form before cleavage into mature caspase 8 (Hughes *et al*, 2009; Dickens *et al*, 2012a), a process that is critically regulated by the catalytically inactive caspase 8 homologue, c-FLIP (Hughes *et al*, 2016). The delineation of this process has now been exploited to develop compounds that are able to bind and activate caspase 8 by stabilising the caspase 8 homodimer, thus enabling faster initiation upon triggering by addition of agonistic TRAIL-R1/R2 antibodies or rhTRAIL (Bucur *et al*, 2015). Importantly, these compounds alone do not trigger apoptosis—it is the combination of the two agents which is effective.

Similarly mechanistic studies into BAK activation in the intrinsic pathway revealed that BAK requires an additional dephosphorylation step in the activation process (Fox *et al*, 2010), before the well-characterised interactions with the pro-apoptotic BH3-only proteins and subsequent conformational change that triggers BAK-dependent apoptosis. Further studies revealed that the kinase responsible for maintaining BAK in this inactive conformation was a tyrosine kinase called BMX (Fox and Storey, 2015), which has been reported to be upregulated in numerous cancer types including breast cancer, prostate cancer, bladder cancer as well as others. Knockdown of BMX *in vitro* was shown to lower the apoptotic threshold of cells by converting BAK into a dephosphorylated ‘activation competent' state, which is more prone to activation. These findings provide a strong mechanism-based rationale for the combination of BMX inhibitors, of which many are currently in development, with other chemotherapeutic agents. As single agents, BMX inhibitors may only have a limited effect on apoptosis as dephosphorylation of BAK alone is not sufficient to trigger BAK activation, but in combination with existing cytotoxic agents would be able to significantly sensitise cancer cells to BAK-dependent apoptosis.

The other BCL-2 effector protein BAX is also regulated by inhibitory phosphorylations that are maintained by different pro-survival pathways including AKT and ERK, which keep BAX auto-inhibited in the cytoplasm and unable to shuttle to the mitochondria. Therefore, in an analogous way as was suggested for BAK-dependent apoptosis, treatment of cells with a compound able to modulate BAX phosphorylation converting BAX into a form that facilitates earlier activation, used in combination with another apoptosis inducing agent, would sensitise cells to apoptosis. This was demonstrated in the combination of BMS-345541, which was developed as an IKK inhibitor but is reported to result in decreased BAX pSer 184 and pThr 167, correspondingly lowering the apoptotic threshold of cells and sensitising previously resistant melanoma cells to TRAIL (Berger *et al*, 2013).

Not only understanding what regulates these proteins, that is specific interactions or phosphorylations, but also the timing of these events can be crucial. Phosphorylation of pro-apoptotic BH3-only protein BID by ATM/ATR has recently be reported to be a determining factor as to whether a cell undergoes apoptosis following mitotic arrest (Wang *et al*, 2014). Once in the phosphorylated form in mitosis, BID is more pro-apoptotic making it more dependent on interaction, with BCL-2 family proteins to inhibit it and prevent apoptosis occurring. The phosphorylation of BID therefore ‘primes' the cells for apoptosis. If mitosis is successfully completed, dephoshorylation occurs and sensitivity to apoptosis is reduced. On the basis of this mechanism, cells arrested in mitosis by the addition of microtubule inhibitors, such as Paxlitaxol, combined with BH3 mimetics such as ABT-737 or Navitoclax became significantly sensitised to apoptosis (Shi *et al*, 2011; Colin *et al*, 2015).

An alternative approach to targeting anti-apoptotic proteins directly is to modulate their expression or activity indirectly. Inhibiting the chaperone protein HSP90 has been of interest for many years due to the huge number of client proteins that it is involved in stabilising thereby facilitating their function. Cancer cells have been found to have a higher dependence on HSP90 than normal cells, thus providing the therapeutic window that has been the basis of the development of HSP90 inhibitors. Inhibition of HSP90 with any of the agents that are currently in clinical trials results in the degradation of key client proteins involved in the progression of the tumour. Of relevance to this review, there are several apoptotic proteins that require HSP90s chaperone activity; these include MCL-1, FLIP, BID and cIAP1. Treatment of colorectal cancer cells and mesothelioma cells with HSP90 inhibitors resulted in rapid degradation of MCL-1 protein specifically in the cancer cells, causing a sensitisation to TRAIL (Lee *et al*, 2015) and ABT-737 (Busacca *et al*, 2015), respectively. Therefore, the combination of HSP90 inhibitors with different pro-apoptotic agents needs to be further investigated, both to overcome acquired resistance and to decrease the apoptotic threshold of cells to sensitise cells to existing chemotherapeutic agents.

Modulation of cancer cell metabolism is another indirect method of sensitising cells to apoptosis. Tumour cells are known to be much more dependent on aerobic glycolysis for metabolism, a phenomenon known as the ‘Warburg effect' (Warburg, 1956). Metabolic switching can therefore be exploited to target cancer cells using agents such as 2-deoxyglucose (2-DG), which interestingly as well as inhibiting glycolysis induces downregulation of MCL-1 levels with no change to BAK expression, suggesting that BAK activation would be increased in the absence of a key anti-apoptotic protein that binds and restrains its activation. In agreement with this, exposure of MCL cells to 2-DG enhanced the sensitivity of these cells to TRAIL-induced apoptosis (Robinson *et al*, 2012). Moreover, in non-Hodgkin's lymphoma (NHL), the effects of 2-DG alone could be further potentiated by the addition of the BH3 mimetic ABT-737 that targets the remaining BCL-2 family members, thereby sensitising NHL cells to both BAK- and BAX-dependent apoptosis (Meynet *et al*, 2012). Although the effects of 2-DG treatment are likely to be tumour cell specific, there are documented changes to protein translation pathways (reviewed in MacFarlane *et al*, 2012) that require further investigation in order to understand the detailed mechanism involved in the observed phenotypes and to understand the complex interactions that occur between metabolism and protein translation to enable any combination studies to be mechanistically driven.

## Future perspectives

As more and more detailed studies into apoptotic signalling pathways and the proteins that regulate these pathways are published, the complexity of the cellular balance between life and death is revealed. It is clear that mechanistic insight must guide the way in which drug combinations are trialled. Furthermore, with numerous examples of off-target toxicity (and more recently ‘on-target' adverse effects) halting drug development, perhaps when drugs are being used to alter the balance of signalling pathways, target modulation should be more often used as a guide in clinical trials rather than the current belt and braces approach using maximum tolerated dose. By utilising strategies to lower the apoptotic signalling threshold of the tumour cell, lower doses of existing cytotoxic agents could be used resulting in better tolerance of often harsh chemotherapeutic regimes. In order to achieve this, however, it is essential that accurate diagnostic analysis of primary tumours is available so that the correct combinations of agents can be used to achieve a positive patient outcome. Furthermore, appropriate pre-clinical models need to be used to test these novel drug combinations, including the use of primary tissue in organotypic explant models as well as models that are able to better predict potentially limiting toxicity. However, utilising mechanistic insight into both the regulation and initiation of apoptotic as well as the non-apoptotic functions of those proteins under investigation as anti-cancer drug targets must be the foundation on which combination therapies are developed in order to reduce both on-target adverse effects and off-target toxicities.

## References

[bib1] Amaravadi RK, Schilder RJ, Martin LP, Levin M, Graham MA, Weng DE, Adjei AA (2015) A phase 1 study of the SMAC-mimetic birinapant in adults with refractory solid tumors or lymphoma. Mol Cancer Ther 14: 2569–2575. 26333381 10.1158/1535-7163.MCT-15-0475

[bib2] Belmar J, Fesik SW (2015) Small molecule Mcl-1 inhibitors for the treatment of cancer. Pharmacol Ther 145: 76–84. 25172548 10.1016/j.pharmthera.2014.08.003 PMC4340597

[bib3] Berger A, Quast SA, Plotz M, Kammermeier A, Eberle J (2013) Sensitization of melanoma cells for TRAIL-induced apoptosis by BMS-345541 correlates with altered phosphorylation and activation of Bax. Cell Death Dis 4: e477. 23348591 10.1038/cddis.2012.198 PMC3563986

[bib4] Bucur O, Gaidos G, Yatawara A, Pennarun B, Rupasinghe C, Roux J, Andrei S, Guo B, Panaitiu A, Pellegrini M, Mierke DF, Khosravi-Far R (2015) A novel caspase 8 selective small molecule potentiates TRAIL-induced cell death. Sci Rep 5: 9893. 25962125 10.1038/srep09893 PMC4426715

[bib5] Busacca S, Law EW, Powley IR, Proia DA, Sequeira M, Le Quesne J, Klabatsa A, Edwards JM, Matchett KB, Luo JL, Pringle JH, El-Tanani M, MacFarlane M, Fennell DA (2015) Resistance to HSP90 inhibition involving loss of MCL1 addiction. Oncogene 35(12): 1483–1492. 26096930 10.1038/onc.2015.213 PMC4819782

[bib6] Colin DJ, Hain KO, Allan LA, Clarke PR (2015) Cellular responses to a prolonged delay in mitosis are determined by a DNA damage response controlled by Bcl-2 family proteins. Open Biol 5(3): 140156. 25761368 10.1098/rsob.140156 PMC4389791

[bib7] Dickens LS, Boyd RS, Jukes-Jones R, Hughes MA, Robinson GL, Fairall L, Schwabe JW, Cain K, MacFarlane M (2012a) A death effector domain chain DISC model reveals a crucial role for caspase-8 chain assembly in mediating apoptotic cell death. Mol Cell 47(2): 291–305. 22683266 10.1016/j.molcel.2012.05.004 PMC3477315

[bib8] Dickens LS, Powley IR, Hughes MA, MacFarlane M (2012b) The ‘complexities' of life and death: death receptor signalling platforms. Exp Cell Res 318(11): 1269–1277. 22542855 10.1016/j.yexcr.2012.04.005

[bib9] Dyer MJ, MacFarlane M, Cohen GM (2007) Barriers to effective TRAIL-targeted therapy of malignancy. J Clin Oncol 25(28): 4505–4506. 17906217 10.1200/JCO.2007.13.1011

[bib10] Fox JL, Ismail F, Azad A, Ternette N, Leverrier S, Edelmann MJ, Kessler BM, Leigh IM, Jackson S, Storey A (2010) Tyrosine dephosphorylation is required for Bak activation in apoptosis. EMBO J 29(22): 3853–3868. 20959805 10.1038/emboj.2010.244 PMC2989102

[bib11] Fox JL, Storey A (2015) BMX negatively regulates BAK function, thereby increasing apoptotic resistance to chemotherapeutic drugs. Cancer Res 75(7): 1345–1355. 25649765 10.1158/0008-5472.CAN-14-1340 PMC4384990

[bib12] Fresquet V, Rieger M, Carolis C, Garcia-Barchino MJ, Martinez-Climent JA (2014) Acquired mutations in BCL2 family proteins conferring resistance to the BH3 mimetic ABT-199 in lymphoma. Blood 123(26): 4111–4119. 24786774 10.1182/blood-2014-03-560284

[bib13] Ganten TM, Koschny R, Sykora J, Schulze-Bergkamen H, Buchler P, Haas TL, Schader MB, Untergasser A, Stremmel W, Walczak H (2006) Preclinical differentiation between apparently safe and potentially hepatotoxic applications of TRAIL either alone or in combination with chemotherapeutic drugs. Clin Cancer Res 12(8): 2640–2646. 16638878 10.1158/1078-0432.CCR-05-2635

[bib14] Han B, Park D, Li R, Xie M, Owonikoko TK, Zhang G, Sica GL, Ding C, Zhou J, Magis AT, Chen ZG, Shin DM, Ramalingam SS, Khuri FR, Curran WJ, Deng X (2015) Small-molecule Bcl2 BH4 antagonist for lung cancer therapy. Cancer Cell 27(6): 852–863. 26004684 10.1016/j.ccell.2015.04.010 PMC4470473

[bib15] Hanahan D, Weinberg RA (2011) Hallmarks of cancer: the next generation. Cell 144(5): 646–674. 21376230 10.1016/j.cell.2011.02.013

[bib16] Haselmann V, Kurz A, Bertsch U, Hubner S, Olempska-Muller M, Fritsch J, Hasler R, Pickl A, Fritsche H, Annewanter F, Engler C, Fleig B, Bernt A, Roder C, Schmidt H, Gelhaus C, Hauser C, Egberts JH, Heneweer C, Rohde AM, Boger C, Knippschild U, Rocken C, Adam D, Walczak H, Schutze S, Janssen O, Wulczyn FG, Wajant H, Kalthoff H, Trauzold A (2014) Nuclear death receptor TRAIL-R2 inhibits maturation of let-7 and promotes proliferation of pancreatic and other tumor cells. Gastroenterology 146(1): 278–290. 24120475 10.1053/j.gastro.2013.10.009

[bib17] Hughes MA, Harper N, Butterworth M, Cain K, Cohen GM, MacFarlane M (2009) Reconstitution of the death-inducing signaling complex reveals a substrate switch that determines CD95-mediated death or survival. Mol Cell 35(3): 265–279. 19683492 10.1016/j.molcel.2009.06.012

[bib18] Hughes MA, Powley IR, Jukes-Jones R, Horn S, Feoktistova M, Fairall L, Schwabe JW, Leverkus M, Cain K, MacFarlane M (2016) Co-operative and hierarchical binding of c-FLIP and Caspase-8: a unified model defines how c-FLIP isoforms differentially control cell fate. Mol Cell 61(6): 834–849. 26990987 10.1016/j.molcel.2016.02.023 PMC4819448

[bib19] Ichim G, Lopez J, Ahmed SU, Muthalagu N, Giampazolias E, Delgado ME, Haller M, Riley JS, Mason SM, Athineos D, Parsons MJ, van de Kooij B, Bouchier-Hayes L, Chalmers AJ, Rooswinkel RW, Oberst A, Blyth K, Rehm M, Murphy DJ, Tait SW (2015) Limited mitochondrial permeabilization causes DNA damage and genomic instability in the absence of cell death. Mol Cell 57(5): 860–872. 25702873 10.1016/j.molcel.2015.01.018 PMC4352766

[bib20] Infante JR, Dees EC, Olszanski AJ, Dhuria SV, Sen S, Cameron S, Cohen RB (2014) Phase I dose-escalation study of LCL161, an oral inhibitor of apoptosis proteins inhibitor, in patients with advanced solid tumors. J Clin Oncol 32(28): 3103–3110. 25113756 10.1200/JCO.2013.52.3993

[bib21] Jeon YJ, Middleton J, Kim T, Lagana A, Piovan C, Secchiero P, Nuovo GJ, Cui R, Joshi P, Romano G, Di Leva G, Lee BK, Sun HL, Kim Y, Fadda P, Alder H, Garofalo M, Croce CM (2015) A set of NF-kappaB-regulated microRNAs induces acquired TRAIL resistance in lung cancer. Proc Natl Acad Sci USA 112(26): E3355–E3364. 26080425 10.1073/pnas.1504630112 PMC4491797

[bib22] Jo M, Kim TH, Seol DW, Esplen JE, Dorko K, Billiar TR, Strom SC (2000) Apoptosis induced in normal human hepatocytes by tumor necrosis factor-related apoptosis-inducing ligand. Nat Med 6(5): 564–567. 10802713 10.1038/75045

[bib23] Kim MS, Kim SS, Yoo NJ, Lee SH (2012) Rare somatic mutation of pro-apoptotic BAX and BAK genes in common human cancers. Tumori 98(6): 149e–151ee. 10.1177/030089161209800625 23389372

[bib24] Lee DH, Sung KS, Bartlett DL, Kwon YT, Lee YJ (2015) HSP90 inhibitor NVP-AUY922 enhances TRAIL-induced apoptosis by suppressing the JAK2-STAT3-Mcl-1 signal transduction pathway in colorectal cancer cells. Cell Signal 27(2): 293–305. 25446253 10.1016/j.cellsig.2014.11.013 PMC4276460

[bib25] Lee SH, Shin MS, Kim HS, Lee HK, Park WS, Kim SY, Lee JH, Han SY, Park JY, Oh RR, Kang CS, Kim KM, Jang JJ, Nam SW, Lee JY, Yoo NJ (2001) Somatic mutations of TRAIL-receptor 1 and TRAIL-receptor 2 genes in non-Hodgkin's lymphoma. Oncogene 20(3): 399–403. 11313970 10.1038/sj.onc.1204103

[bib26] Leverkus M, Neumann M, Mengling T, Rauch CT, Brocker EB, Krammer PH, Walczak H (2000) Regulation of tumor necrosis factor-related apoptosis-inducing ligand sensitivity in primary and transformed human keratinocytes. Cancer Res 60(3): 553–559. 10676636

[bib27] Leverson JD, Zhang H, Chen J, Tahir SK, Phillips DC, Xue J, Nimmer P, Jin S, Smith M, Xiao Y, Kovar P, Tanaka A, Bruncko M, Sheppard GS, Wang L, Gierke S, Kategaya L, Anderson DJ, Wong C, Eastham-Anderson J, Ludlam MJ, Sampath D, Fairbrother WJ, Wertz I, Rosenberg SH, Tse C, Elmore SW, Souers AJ (2015) Potent and selective small-molecule MCL-1 inhibitors demonstrate on-target cancer cell killing activity as single agents and in combination with ABT-263 (navitoclax). Cell Death Dis 6: e1590. 25590800 10.1038/cddis.2014.561 PMC4669759

[bib28] Lim B, Allen JE, Prabhu VV, Talekar MK, Finnberg NK, El-Deiry WS (2015) Targeting TRAIL in the treatment of cancer: new developments. Expert Opin Ther Targets 19(9): 1171–1185. 26004811 10.1517/14728222.2015.1049838

[bib29] Lovric MM, Hawkins CJ (2010) TRAIL treatment provokes mutations in surviving cells. Oncogene 29(36): 5048–5060. 20639907 10.1038/onc.2010.242 PMC2997681

[bib30] MacFarlane M, Kohlhaas SL, Sutcliffe MJ, Dyer MJ, Cohen GM (2005) TRAIL receptor-selective mutants signal to apoptosis via TRAIL-R1 in primary lymphoid malignancies. Cancer Res 65(24): 11265–11270. 16357130 10.1158/0008-5472.CAN-05-2801

[bib31] MacFarlane M, Robinson GL, Cain K (2012) Glucose—a sweet way to die: metabolic switching modulates tumor cell death. Cell Cycle 11(21): 3919–3925. 22983094 10.4161/cc.21804 PMC3507486

[bib32] Meynet O, Beneteau M, Jacquin MA, Pradelli LA, Cornille A, Carles M, Ricci JE (2012) Glycolysis inhibition targets Mcl-1 to restore sensitivity of lymphoma cells to ABT-737-induced apoptosis. Leukemia 26(5): 1145–1147. 22076465 10.1038/leu.2011.327

[bib33] Montero J, Sarosiek KA, DeAngelo JD, Maertens O, Ryan J, Ercan D, Piao H, Horowitz NS, Berkowitz RS, Matulonis U, Janne PA, Amrein PC, Cichowski K, Drapkin R, Letai A (2015) Drug-induced death signaling strategy rapidly predicts cancer response to chemotherapy. Cell 160(5): 977–989. 25723171 10.1016/j.cell.2015.01.042 PMC4391197

[bib34] Ni Chonghaile T, Sarosiek KA, Vo TT, Ryan JA, Tammareddi A, Moore Vdel G, Deng J, Anderson KC, Richardson P, Tai YT, Mitsiades CS, Matulonis UA, Drapkin R, Stone R, Deangelo DJ, McConkey DJ, Sallan SE, Silverman L, Hirsch MS, Carrasco DR, Letai A (2011) Pretreatment mitochondrial priming correlates with clinical response to cytotoxic chemotherapy. Science 334(6059): 1129–1133. 22033517 10.1126/science.1206727 PMC3280949

[bib35] Papadopoulos KP, Isaacs R, Billic S, Kentsch K, Huet HA, Hofmann M, Rasco D, Kundamal N, Tang Z, Cooksey J, Mahipal A (2015) Unexpected hepatotoxicity in a phase I study of TAS266, a novel tetravalent agonistic Nanobody® targeting the DR5 receptor. Cancer Chemother Pharmacol 75: 887–895. 25721064 10.1007/s00280-015-2712-0

[bib36] Perciavalle RM, Stewart DP, Koss B, Lynch J, Milasta S, Bathina M, Temirov J, Cleland MM, Pelletier S, Schuetz JD, Youle RJ, Green DR, Opferman JT (2012) Anti-apoptotic MCL-1 localizes to the mitochondrial matrix and couples mitochondrial fusion to respiration. Nat Cell Biol 14(6): 575–583. 22544066 10.1038/ncb2488 PMC3401947

[bib37] Petersen SL, Wang L, Yalcin-Chin A, Li L, Peyton M, Minna J, Harran P, Wang X (2007) Autocrine TNFalpha signaling renders human cancer cells susceptible to Smac-mimetic-induced apoptosis. Cancer Cell 12(5): 445–456. 17996648 10.1016/j.ccr.2007.08.029 PMC3431210

[bib38] Roberts AW, Davids MS, Pagel JM, Kahl BS, Puvvada SD, Gerecitano JF, Kipps TJ, Anderson MA, Brown JR, Gressick L, Wong S, Dunbar M, Zhu M, Desai MB, Cerri E, Heitner Enschede S, Humerickhouse RA, Wierda WG, Seymour JF (2016) Targeting BCL2 with venetoclax in relapsed chronic lymphocytic leukemia. N Engl J Med 374(4): 311–322. 26639348 10.1056/NEJMoa1513257 PMC7107002

[bib39] Robinson GL, Dinsdale D, MacFarlane M, Cain K (2012) Switching from aerobic glycolysis to oxidative phosphorylation modulates the sensitivity of mantle cell lymphoma cells to TRAIL. Oncogene 31(48): 4996–5006. 22310286 10.1038/onc.2012.13

[bib40] Shi J, Zhou Y, Huang HC, Mitchison TJ (2011) Navitoclax (ABT-263) accelerates apoptosis during drug-induced mitotic arrest by antagonizing Bcl-xL. Cancer Res 71(13): 4518–4526. 21546570 10.1158/0008-5472.CAN-10-4336 PMC3129452

[bib41] Sochalska M, Tuzlak S, Egle A, Villunger A (2015) Lessons from gain- and loss-of-function models of pro-survival Bcl2 family proteins: implications for targeted therapy. FEBS J 282(5): 834–849. 25559680 10.1111/febs.13188 PMC4562365

[bib42] Tchoghandjian A, Jennewein C, Eckhardt I, Momma S, Figarella-Branger D, Fulda S (2014) Smac mimetic promotes glioblastoma cancer stem-like cell differentiation by activating NF-kappaB. Cell Death Differ 21(5): 735–747. 24488095 10.1038/cdd.2013.200 PMC3978303

[bib43] Tchoghandjian A, Jennewein C, Eckhardt I, Rajalingam K, Fulda S (2013) Identification of non-canonical NF-kappaB signaling as a critical mediator of Smac mimetic-stimulated migration and invasion of glioblastoma cells. Cell Death Dis 4: e564. 23538445 10.1038/cddis.2013.70 PMC3615728

[bib44] Thomas RL, Roberts DJ, Kubli DA, Lee Y, Quinsay MN, Owens JB, Fischer KM, Sussman MA, Miyamoto S, Gustafsson AB (2013) Loss of MCL-1 leads to impaired autophagy and rapid development of heart failure. Genes Dev 27(12): 1365–1377. 23788623 10.1101/gad.215871.113 PMC3701192

[bib45] Varadarajan S, Butterworth M, Wei J, Pellecchia M, Dinsdale D, Cohen GM (2013) Sabutoclax (BI97C1) and BI112D1, putative inhibitors of MCL-1, induce mitochondrial fragmentation either upstream of or independent of apoptosis. Neoplasia 15(5): 568–578. 23633928 10.1593/neo.13230 PMC3638359

[bib46] Varfolomeev E, Blankenship JW, Wayson SM, Fedorova AV, Kayagaki N, Garg P, Zobel K, Dynek JN, Elliott LO, Wallweber HJ, Flygare JA, Fairbrother WJ, Deshayes K, Dixit VM, Vucic D (2007) IAP antagonists induce autoubiquitination of c-IAPs, NF-kappaB activation, and TNFalpha-dependent apoptosis. Cell 131(4): 669–681. 18022362 10.1016/j.cell.2007.10.030

[bib47] Vela L, Marzo I (2015) Bcl-2 family of proteins as drug targets for cancer chemotherapy: the long way of BH3 mimetics from bench to bedside. Curr Opin Pharmacol 23: 74–81. 26079328 10.1016/j.coph.2015.05.014

[bib48] Vince JE, Wong WW, Khan N, Feltham R, Chau D, Ahmed AU, Benetatos CA, Chunduru SK, Condon SM, McKinlay M, Brink R, Leverkus M, Tergaonkar V, Schneider P, Callus BA, Koentgen F, Vaux DL, Silke J (2007) IAP antagonists target cIAP1 to induce TNFalpha-dependent apoptosis. Cell 131(4): 682–693. 18022363 10.1016/j.cell.2007.10.037

[bib49] Vo TT, Ryan J, Carrasco R, Neuberg D, Rossi DJ, Stone RM, Deangelo DJ, Frattini MG, Letai A (2012) Relative mitochondrial priming of myeloblasts and normal HSCs determines chemotherapeutic success in AML. Cell 151(2): 344–355. 23063124 10.1016/j.cell.2012.08.038 PMC3534747

[bib50] Volkmann X, Fischer U, Bahr MJ, Ott M, Lehner F, MacFarlane M, Cohen GM, Manns MP, Schulze-Osthoff K, Bantel H (2007) Increased hepatotoxicity of tumor necrosis factor-related apoptosis-inducing ligand in diseased human liver. Hepatology 46(5): 1498–1508. 17705261 10.1002/hep.21846

[bib51] von Karstedt S, Conti A, Nobis M, Montinaro A, Hartwig T, Lemke J, Legler K, Annewanter F, Campbell AD, Taraborrelli L, Grosse-Wilde A, Coy JF, El-Bahrawy MA, Bergmann F, Koschny R, Werner J, Ganten TM, Schweiger T, Hoetzenecker K, Kenessey I, Hegedus B, Bergmann M, Hauser C, Egberts JH, Becker T, Rocken C, Kalthoff H, Trauzold A, Anderson KI, Sansom OJ, Walczak H (2015) Cancer cell-autonomous TRAIL-R signaling promotes KRAS-driven cancer progression, invasion, and metastasis. Cancer Cell 27(4): 561–573. 25843002 10.1016/j.ccell.2015.02.014 PMC6591140

[bib52] Wang P, Lindsay J, Owens TW, Mularczyk EJ, Warwood S, Foster F, Streuli CH, Brennan K, Gilmore AP (2014) Phosphorylation of the proapoptotic BH3-only protein bid primes mitochondria for apoptosis during mitotic arrest. Cell Rep 7(3): 661–671. 24767991 10.1016/j.celrep.2014.03.050 PMC4022835

[bib53] Warburg O (1956) On respiratory impairment in cancer cells. Science 124(3215): 269–270. 13351639

[bib54] Yu L, Liu S (2013) Autophagy contributes to modulating the cytotoxicities of Bcl-2 homology domain-3 mimetics. Semin Cancer Biol 23(6 Pt B): 553–560. 24012660 10.1016/j.semcancer.2013.08.008

